# Modified Balance Error Scoring System (M-BESS) test scores in athletes wearing protective equipment and cleats

**DOI:** 10.1136/bmjsem-2016-000117

**Published:** 2016-05-13

**Authors:** Aftab Mohammad Azad, Saad Al Juma, Junaid Ahmad Bhatti, J Scott Delaney

**Affiliations:** 1 Department of Emergency Medicine, McGill University Health Centre, Montreal, Québec, Canada; 2 Department of Sport Medicine, McGill University, Montreal, Québec, Canada; 3 Department of Accident and Emergency, Hamad Medical Corporation, Doha, Qatar; 4 Sunnybrook Research Institute, Toronto, Ontario, Canada

**Keywords:** Concussion, Soccer, Muscle imbalance, American football

## Abstract

**Background:**

Balance testing is an important part of the initial concussion assessment. There is no research on the differences in Modified Balance Error Scoring System (M-BESS) scores when tested in real world as compared to control conditions.

**Objective:**

To assess the difference in M-BESS scores in athletes wearing their protective equipment and cleats on different surfaces as compared to control conditions.

**Methods:**

This cross-sectional study examined university North American football and soccer athletes. Three observers independently rated athletes performing the M-BESS test in three different conditions: (1) wearing shorts and T-shirt in bare feet on firm surface (control); (2) wearing athletic equipment with cleats on FieldTurf; and (3) wearing athletic equipment with cleats on firm surface. Mean M-BESS scores were compared between conditions.

**Results:**

60 participants were recruited: 39 from football (all males) and 21 from soccer (11 males and 10 females). Average age was 21.1 years (SD=1.8). Mean M-BESS scores were significantly lower (p<0.001) for cleats on FieldTurf (mean=26.3; SD=2.0) and for cleats on firm surface (mean=26.6; SD=2.1) as compared to the control condition (mean=28.4; SD=1.5). Females had lower scores than males for cleats on FieldTurf condition (24.9 (SD=1.9) vs 27.3 (SD=1.6), p=0.005). Players who had taping or bracing on their ankles/feet had lower scores when tested with cleats on firm surface condition (24.6 (SD=1.7) vs 26.9 (SD=2.0), p=0.002).

**Conclusions:**

Total M-BESS scores for athletes wearing protective equipment and cleats standing on FieldTurf or a firm surface are around two points lower than M-BESS scores performed on the same athletes under control conditions.

**Strengths and limitations of this study**

- This study shows that university soccer athletes wearing full equipment and cleats tested on FieldTurf or a firm surface make around two more errors on Modified Balance Error Scoring System (M-BESS) testing as compared to control testing performed barefoot in shorts and a T-shirt on a firm surface.
- Different individual factors such as gender and the wearing of a brace or tape at the ankles/feet area may affect total M-BESS scores in different field conditions.
- These findings support the use of M-BESS in field settings for screening concussion if deviations from the baseline are seen.
- Findings may not be generalisable to athletes from other sports wearing a different set of equipment.

## Introduction

Concussion is a brain injury characterised by an alteration in cerebral function caused by a direct acceleration or deceleration force transmitted to a freely mobile head.^1^ ^2^ It is estimated that between 1.6 and 3.8 million sports-related concussions occur each year in the USA.^3–5^ It is a common injury in contact sports such as soccer and North American football.^4^ Consensus guidelines recommend removing any player who is exhibiting signs or symptoms of a concussion for evaluation.^1^ ^2^ ^6^ Making the diagnosis of a concussion is not always easy, as players can deny symptoms^7^ and neurocognitive testing may be similar, or only slightly different from baseline.^8^ The physical and traditional neurological examinations are often normal. The Balance Error Scoring System (BESS) was developed as an objective test to assess concussed athletes.^9^ ^10^ This test has been found to be a useful physical examination tool to help differentiate concussed from non-concussed athletes, especially within the first few days following injury.^11^

Examination of a potentially concussed athlete should ideally occur in a quiet space, removed from the distractions of the athletic environment.^12^ The diagnosis is often made more difficult by the fact that this assessment must be done in a timely fashion. To overcome the challenges of screening for concussion on the sidelines, a number of clinical assessment tools have been proposed.^1^ ^6^ ^13^ The Sport Concussion Assessment Tool versions 2 and 3 (SCAT-2 and SCAT-3) are commonly used tools that assess symptoms, physical, neurological, cognitive, balance and coordination examinations.^6^ ^13^ The balance examination is a critical section of SCAT-2 and SCAT-3. While the traditional BESS testing is completed on a firm surface followed by testing on a foam surface, the modified version of BESS (M-BESS) included in SCAT-2 and SCAT-3 only assesses balance on a firm surface.^14–16^ The M-BESS requires balancing in three stances, that is, in double leg, single leg and tandem gait performed with eyes closed, hands placed on hips and standing barefoot on a firm surface.^17^ Balancing in each of the three stances is assessed with a maximum score of 10 points each. Mistakes are subtracted from the score, making the maximum total M-BESS score of 30.^17^ The normative scores for M-BESS have been published recently,^15^ ^16^ but clinical judgment prevails as the gold standard for diagnosing concussion.^10^ ^18^ To date, definitive research data are lacking on what absolute M-BESS scores reliably help rule in, or rule out, a concussion.^19^ Similarly, the literature on factors affecting M-BESS scores is unavailable. Nonetheless, studies on the traditional version of BESS test performed on firm and foam surfaces indicate that performances can be influenced by gender (females better than males), training, bracing, injury, exercise, fatigue and time since injury.^20^ ^21^

While some have suggested that M-BESS scores are reliable in athletes wearing athletic gear and cleats,^19^ to date, studies have not evaluated the M-BESS performance in athletes under these conditions.^22^ ^23^ The knowledge gained by such a study might help sport medicine professionals who have to examine athletes during a game or practice when it is not feasible to have an athlete remove all of their gear and cleats.^19^ Therefore, the objective of this study is to assess the difference in M-BESS test scores in athletes wearing their protective equipment with cleats in two field conditions as compared to the traditional testing performed in bare feet on a firm surface while wearing shorts and a T-shirt.

## Methods

### Setting

The study was conducted in the sport medicine clinic at McGill University, Montreal, Québec, Canada, during the 2014 varsity season. The university has male and female varsity soccer teams and a male varsity North American football team. The age range of players usually varies from 18 to 30 years. Each team is followed by a team of therapists/trainers, a sport medicine fellow and a supervising attending physician from the Department of Sport Medicine. Ethics approval was obtained from the Research Ethics Board of McGill University Health Centre.

### Study design

In this cross-sectional study, three observers assessed M-BESS performances in male and female players under three conditions: (1) no protective equipment but wearing shorts and T-shirts in bare feet on a firm surface (control condition); (2) full protective equipment with cleats on FieldTurf; and (3) full protective equipment with cleats on firm surface. Football players removed their helmets for the M-BESS evaluations. FieldTurf is the surface used for all varsity games for football and soccer at McGill University. It is a synthetic surface which consists of polyethylene blend fibres with an infill bottom layer of sand, a middle layer mixture of sand and cryogenic rubber and a top layer of rubber. The fibres are meant to replicate blades of grass, while the infill acts as a cushion. A hard firm surface was used as the other testing condition, as this is the surface normally used for control measurements. It is also usually readily available to most sport medicine professionals during practice or game conditions, either in the locker room or close to the sidelines.

The sample size estimation (n>37) was based on the assumption that M-BESS performances with and without cleats would be highly concordant (intraclass correlation coefficient (ICC) ≈ 0.80 with 95% CI of 0.20) with type I error set at 0.05% and study power at 95%.

### Participants

Sampling was convenience based. This study included athletes from varsity football and soccer teams between the ages of 18 and 30 years. Those who were unfit to practise or play due to any injury or illness (eg, musculoskeletal injury, concussion, infectious illness) or had any known balance or vestibular problems were excluded. All participants signed informed consent and received a monetary compensation ($20) for their time.

A total of 60 players were included in this convenience sampling-based study (table 1). Players were called by their coaches during the practice sessions to participate in this study. The study included athletes from varsity football (n=39) and soccer teams (n=21). No players approached refused to participate or were excluded because of current illness, injury (eg, musculoskeletal injury, concussion, infectious illness) or known balance or vestibular problems. For soccer athletes, 10 were females and 11 were males. Average age for the 60 athletes studied was 21.1 years (SD=1.8; range 18–25 years). Average body mass index (BMI) was 27.9 kg/m^2^ (SD=5.0). A significant proportion of players had a history of lower extremity injury (40.0%, n=24) or past diagnosed concussion (31.7%, n=19), but were not suffering from any of the condition at the time of testing. Approximately one in seven players (13.3%, n=8) had bracing of the ankles/feet and one player (1.7%) had tape wrapped at their ankles/feet during field testing. No players had bracing or taping of their ankles/feet during control testing.

**Table 1:** Characteristics of study participants

	Athletes, (n)	Per cent
Gender		
Male	50	83.3
Female	10	16.7
Players		
Soccer	21	35.0
Football	39	65.0
Tape*	1	1.7
Brace*	8	13.3
History of concussion	19	31.7
History of lower extremity injury	24	40.0
	**Mean**	**SD**
Age	21.1	1.8
Body mass index	27.9	5.0

*Taping or bracing was only on the ankle/foot area during field testing.

### Measures

Three observers (two fellows in sport medicine and one senior resident in emergency medicine) assessed M-BESS performance in three stances, in three conditions, independently of each other. One author, a sport medicine physician with over 20 years' experience, led an information session on proper M-BESS assessment prior to data collection to ensure uniform knowledge and standardised procedures for the M-BESS assessment. Before each test, the same set of instructions for the M-BESS was read aloud to each athlete, with an opportunity to ask questions or for clarification prior to beginning the test. The control condition was evaluated in a sport medicine clinic, while the other field conditions requiring the athletes to wear their protective equipment occurred on separate days on the sidelines during, or immediately after, team practices.

In each stance, the three observers counted the errors in deviations from the proper stance, that is, moving hands off of iliac crests, opening eyes, a step/stumble or fall, abduction or flexion of the hip beyond 30°, lifting forefoot or heel off testing surface and remaining out of the proper testing position for >5 s. Only one error was counted when multiple errors occurred at the same time. The number of errors in each stance was subtracted from a score of 10 for each of the three stances. The maximum total score for each testing condition was 30. Information about players' age, gender, height, weight, team membership (football or soccer), use of brace or taping, history of lower extremity injury and any past diagnosed concussions was recorded on a separate sheet.

### Analyses

All the information gathered from testing was later entered on a spreadsheet by one of the investigators. A total of 10% of entries were verified by a second investigator with expected error rate of <1%. Mean M-BESS performance scores for each stance and total scores were computed by averaging scores by three observers. Paired t tests were used to assess differences between conditions as: (1) full protective equipment with cleats on FieldTurf versus control; (2) full protective equipment with cleats on firm surface versus control; and (3) full protective equipment with cleats on FieldTurf versus full protective equipment with cleats on firm surface. The above differences in M-BESS scores were also assessed for various player characteristics—for example, by age, BMI categories, team, taping or bracing of ankles/feet, history of lower limb injury and concussion. Further, individual differences for M-BESS, for example, by age, for each of the field conditions were assessed using Student's t test. Last, we assessed the interobserver reliability for three conditions by computing ICCs.^22^

## Results

The mean total M-BESS scores and mean scores for the individual position components for each of the three conditions tested are listed in table 2. Single-leg stance, tandem-leg stance and total M-BESS performance scores were significantly different (p<0.001) for both field testing conditions compared to control conditions. Double-leg stance for all conditions tested was almost identical as only two errors occurred in one athlete during the full equipment with cleats on FieldTurf M-BESS testing. The difference in total M-BESS scores was roughly two points lower for athletic equipment with cleats on FieldTurf versus control and for athletic equipment with cleats on firm surface versus control. The mean total M-BESS scores for athletic equipment with cleats on FieldTurf were 26.3 (SD=2.0), athletic equipment with cleats on firm surface were 26.6 (SD=2.1) and control were 28.4 (SD=1.5). There were no differences between the two different field conditions for single-leg stance (p=0.60), tandem-leg stance (p=0.57) and total M-BESS performance scores (p=0.26).

**Table 2:** Mean scores for Modified Balance Error Scoring System (M-BESS) by three observers for three different testing conditions

	Mean	SD
Barefoot on firm surface (control)		
Double legs	10.0	0
Single leg	8.7	1.3
Tandem	9.7	0.5
Total	28.4	1.5
Cleats on FieldTurf*†		
Double legs	9.9	0.4
Single leg	7.2	1.4
Tandem	9.2	1.2
Total	26.3	2.0
Cleats on firm surface*†		
Double legs	10.0	0
Single leg	7.3	1.7
Tandem	9.3	0.9
Total	26.6	2.1

N=60 athletes tested.

*Wearing full protective equipment with cleats.

†Football players removed their helmets for the M-BESS evaluation.

Table 3 shows the effects of player characteristics on mean total M-BESS scores for control versus the two field conditions. Overall, the significant differences between control and the two field conditions persisted in almost all player characteristics except two conditions. For the subset of players with a BMI≥30 kg/m^2^ (who were all male football players), there was no difference between control testing and both field testing conditions for mean total M-BESS scores. Also, male soccer players had no statistical difference in mean total M-BESS scores between control testing and testing with athletic equipment and cleats on firm surface.

**Table 3:** Association of player characteristics with differences in total Modified Balance Error Scoring System (M-BESS) scores for three different testing conditions

		Barefoot on firm surface (control)		Cleats* on FieldTurf		Cleats* on firm surface	
		M-BESS		M-BESS		M-BESS	
	Athletes, (n)	M	SD	M	SD	M	SD
Gender (only soccer players)							
Male	11	29.1	1.2	27.3†	1.6	27.8‡	2.0
Female	10	28.5	1.0	24.9†	1.9	26.2†	1.9
Players (only male players)							
Football§	39	28.2	1.6	26.4†	2.0	26.4†	2.2
Age (years)							
18–21	39	28.4	1.6	26.5†	2.0	26.8†	2.1
22–30	21	28.4	1.3	26.0†	2.1	26.2†	2.1
Body mass index (kg/m^2^)							
<25	24	28.8	1.2	26.1†	2.1	26.8†	2.0
25 to <30	24	28.4	1.4	26.5†	2.1	26.4†	2.3
≥30	12	27.8	1.9	26.3‡	1.9	26.5‡	2.1
Taping or braces¶							
No	51	28.6	1.3	26.5†	2.0	26.9†	2.0
Yes	9	27.6	1.9	25.2†	2.2	24.6†	1.7
History of concussion							
No	41	28.4	1.4	26.1†	2.0	26.5†	2.1
Yes	19	28.5	1.6	26.8†	2.1	26.9†	2.3
History of lower limb injury							
No	36	28.2	1.6	26.5†	1.8	26.8†	2.0
Yes	24	28.8	1.2	26.1†	2.4	26.3†	2.3

p Values refer to differences from control M-BESS values.

*Wearing full protective equipment with cleats.

†Statistically different from control (p<0.01).

‡Statistically different from control (0.01≤p<0.05).

§Football players removed their helmets for the M-BESS evaluation.

¶Taping or bracing was only on the ankle/foot area during field testing.

M, mean.

There was no significant difference in the mean total M-BESS scores between male football and soccer players for all conditions tested (p=0.06 or higher). In fact, we found no significant difference in M-BESS scores between player groups except for two cases. First, female soccer players performed significantly worse than male soccer players for cleats on FieldTurf condition (24.9 vs 27.3, p=0.005). Second, players who had taping or bracing of their ankles/feet had significantly lower scores than other players for cleats on firm surface condition (24.6 vs 26.9, p=0.002).

A moderate-to-high interobserver reliability (0.60≤ICC≤0.75) was observed for total M-BESS scores under three conditions (table 4). The interobserver reliability was higher for barefoot on firm surface condition as compared to the other two field conditions.

**Table 4:** Interobserver (n=3) reliability of Modified Balance Error Scoring System (M-BESS) scores for three different testing conditions

	Intraclass correlation coefficient	95% CIs
Barefoot on firm surface (control)		
Single leg	0.75	0.64 to 0.84
Tandem	0.71	0.56 to 0.81
Total	0.75	0.63 to 0.84
Cleats on FieldTurf*†		
Single leg	0.53	0.36 to 0.68
Tandem	0.52	0.36 to 0.37
Total	0.60	0.44 to 0.73
Cleats on firm surface*†		
Single leg	0.61	0.46 to 0.74
Tandem	0.67	0.54 to 0.79
Total	0.68	0.54 to 0.79

Intraclass correlation coefficients were not estimated for double-leg stance because there were nearly no balancing errors.

*Wearing full protective equipment with cleats.

†Football players removed their helmets for the M-BESS evaluation.

## Discussion

This study quantifies the differences in M-BESS scores that should be expected if the test is performed in real-world field conditions for university athletes playing football or soccer. Findings suggest that, as compared to control testing, mean M-BESS scores were around 2 points lower when performed with protective equipment and cleats on FieldTurf or firm surface.

These findings are relevant for health professionals in sports, perhaps as well as coaches, who both have the responsibility of assuring the safety of the players.^24^ This study extends the scope of M-BESS assessments, and it shows that M-BESS testing is feasible in different field conditions. Findings indicate to what extent scores should be adjusted when evaluating football or soccer athletes wearing protective equipment with cleats on two different surfaces.

While this study did not assess acutely concussed athletes, it suggests that non-concussed athletes tested on FieldTurf or a firm surface wearing their protective equipment and cleats should be expected to make on average two more mistakes during M-BESS testing as compared to control testing. If athletes are to be tested in field conditions during game or practice situations, it would seem prudent to gather baseline scores while they are wearing their protective equipment and cleats either on a firm or their regular playing surface. This would allow sport medicine professionals to compare an athlete's postinjury M-BESS scores with their own baseline score performed under similar conditions and on similar surfaces. These precautions may help in avoiding misinterpretation of M-BESS scores in field conditions that are different from control testing, as was observed in this study.

### Equipment versus cleats

In this study, during field testing conditions, football athletes were wearing shoulder pads, hip pads, thigh pads, a jersey, pants and cleats (helmets were removed for testing), while soccer athletes were wearing only shin pads, a jersey, shorts and cleats. Given the fact there were no significant differences in mean M-BESS scores between football and soccer players for any of the three testing conditions, differences from control conditions for both groups of athletes are likely due to the wearing of cleats during field testing conditions. While football and soccer players practise and play on the same FieldTurf, they do wear different styles of cleats. The type of cleats and length of studs on the cleats were not assessed in this study, but it may be an area of future study.

### Inter-rater reliability

This study noted that inter-rater reliability decreased in conditions other than the control condition. This indicates that sport medicine professionals using M-BESS in field conditions should receive periodic group training to ensure as much homogeneity as possible when interpreting M-BESS errors in athletes wearing protective equipment and cleats in field conditions.

### Limitations

This study has several limitations. First, only players from two sports were recruited. Therefore, findings might not be applicable to other sports using other equipment—for example, lacrosse and baseball.^25^ Second, there were only 10 female soccer players and 11 male soccer players in the study. Any differences between males and females, field testing versus control testing for soccer players, etc, may be better identified by a larger study with more soccer players, and in particular, more females included in the study group. Third, the M-BESS performance was measured during training sessions, which might have different effects on fatigue levels as compared to a competitive match.^23^ The findings might therefore be biased towards observing small differences. Nonetheless, an attempt was made to recruit players towards the end of the training session to account for the effects of fatigue. Fourth, testing was not performed on any natural grass surfaces. FieldTurf is a fairly flat consistent surface, whereas natural grass may be uneven and the firmness may vary under different weather conditions. Fifth, there are a range of values that may be seen in normal testing, in all conditions, as evidenced by our SDs. While a larger study may help to lower SDs and detect differences in the effect of different player characteristics on testing which this study was unable to find, it is likely that a range of normal values will always exist when testing a large group of athletes. While data do exist as to what should constitute an obvious abnormal traditional BESS score done on firm and foam surfaces, similar data values do not exist for the M-BESS scoring system.^19^ As per current practice, the findings suggest the responsibility of interpreting M-BESS scores relies on individual sport medicine professionals. As mentioned previously, having a baseline score under the same field conditions may be helpful when interpreting an individual athlete's postinjury M-BESS scores. Last, these tests were performed on non-concussed athletes. The differences in M-BESS scores in field conditions versus control for concussed athletes have not been determined. While it may be logical to assume that M-BESS scores with significantly higher than a two-point difference between control and field condition testing may be due to other effects, such as a concussion, the fact that a range of normal values exist for M-BESS testing must be taken into consideration.

## Conclusion

This study showed that total M-BESS performance scores in university North American football and soccer athletes wearing protective equipment with cleats in field settings were roughly two points less than the control tests performed in barefoot conditions on a firm surface. These findings may make M-BESS more accessible to sport medicine professionals who often face the reality of not being able to test an injured player in an ideal clinical setting during an ongoing match.
